# A novel near-infrared II viscosity-responsive probe for surgical fluorescence guidance: laboratory investigation in a murine subcutaneous glioma model

**DOI:** 10.3389/fonc.2026.1586263

**Published:** 2026-02-11

**Authors:** Lihao Lin, Tianyang Han, Huizhong Jiang, Yuewei Zhang, Yi Guan

**Affiliations:** 1 Department of Neurosurgery, First Hospital of Jilin University, Changchun, China; 2 Joint Laboratory of Opto-Functional Theranostics in Medicine and Chemistry, First Hospital of Jilin University, Changchun, China; 3 Department of Emergency, First Hospital of Jilin University, Changchun, China; 4 School of Chemistry and Pharmaceutical Engineering, Jilin Institute of Chemical Technology, Jilin, China

**Keywords:** fluorescence guidance, glioma, ICG, NIR-II imaging, surgical navigation, viscosity probes

## Abstract

Distinguishing brain tumor boundaries from surrounding parenchymal tissue with high sensitivity and specificity remains a daunting clinical challenge due to their diffuse nature and proximity to critical brain regions. Currently approved clinical fluorescent contrast agents are insufficient for clearly delineating the margins of gliomas. This study evaluated a novel near-infrared II (NIR-II) viscosity responsive fluorescent probe, POH, for fluorescence guided surgery in glioma models. Compared to commercial dyes, this probe offers advantages such as strong photostability, large Stokes shift, and high responsiveness to tumor tissue. Statistical analysis of postoperative survival and tissue margins demonstrated that the viscosity responsive probe POH exhibited significant advantages over the mainstream NIR contrast agent ICG. The unique chemical structure of this semi-cyanine derivative offers potential advantages for fluorescence guided surgery of gliomas, providing new insights into the design and selection of surgical navigation contrast agents. However, it should be noted that the subcutaneous glioma model used in this study does not replicate the intracranial tumor microenvironment or the impact of the blood-brain barrier. Future studies using orthotopic glioma models are essential to fully evaluate the translational potential of POH.

## Introduction

Gliomas often lack clear boundaries with normal brain tissue, making it challenging for surgeons to completely remove tumor cells without damaging healthy tissue (1–7). Gliomas typically infiltrate adjacent brain tissue, forming diffuse boundaries that traditional imaging techniques struggle to delineate clearly (7–12). In light of this, improved surgical guidance technology is a promising strategy. Fluorescence guided surgery is considered the next generation clinical diagnostic and therapeutic technology, with the near-infrared II window (NIR-II, 1000–1700 nm) receiving widespread attention for its unique penetration depth and low background signal (13–19). NIR-II fluorescence imaging is an emerging and rapidly developing biomedical imaging technology, offering deeper penetration and higher spatial resolution than NIR-I (700–1000 nm) (16, 20–22). To date, various NIR-II fluorescent dyes have been developed, including carbon-based nanomaterials, metal quantum dots, and organic semiconductor polymers (23–25).

Cyanine dyes have been extensively explored in recent years owing to their high molar extinction coefficients, superior fluorescence quantum yields, and flexible chemical structures that allow spectral tunability and functional modification (26–29). Among these, indocyanine green (ICG), an FDA approved NIR-I dye traditionally used for angiography, has become one of the most widely investigated agents for fluorescence-guided surgery (30–34). Building upon its clinical safety and well-characterized pharmacokinetics, Dr. John Y. K. Lee and colleagues developed the “Second-Window ICG (SWIG)” technique, which utilizes delayed high-dose ICG administration to enhance tumor-to-background contrast during glioma resection. Their pioneering studies demonstrated that SWIG allows for reliable intraoperative visualization of high-grade gliomas and shows strong correlation with postoperative gadolinium-enhanced MRI findings (35–37). This strategy significantly broadens the clinical applicability of ICG and highlights the potential of near-infrared fluorescence imaging in neurosurgical navigation. Nevertheless, conventional ICG and related dyes still face limitations in clinical applications, such as shallow tissue penetration, dependence on endoscopic guidance, and reduced performance in tumors with diffuse or ill-defined margins (38–41).

For gliomas, viscosity, defined as the internal friction or resistance to molecular motion within the tumor microenvironment, is a critical physiological parameter. Increased microviscosity in tumor tissues arises from dense cellular packing and altered metabolic activity, which can significantly influence the fluorescence response of viscosity-sensitive probes (42–44). We utilized a viscosity-responsive probe based on a classic twisted intramolecular charge transfer (TICT) semi-cyanine structure for fluorescence-guided surgery. Here, we selected a novel viscosity-responsive probe, POH, based on the classic semi-cyanine structure and compared it with the classic ICG probe in fluorescence-guided surgery on high-grade glioma mouse models. The new probe demonstrated higher Stokes shifts, greater photostability, and stronger fluorescence intensity and imaging contrast in tumor regions than traditional cyanine probes.

We comprehensively evaluated the probe’s biosafety and metabolic profile in a mouse subcutaneous tumor model. By injecting the probe *in situ*, we performed surgical resection followed by closure of the surgical incision on the glioma mouse model at the optimal time point. Professionally trained clinical medical students performed all tumor resections and postoperative wound closures under sterile conditions.

Through a follow-up observation period of about six weeks post-surgery, we found that this treatment regimen significantly outperformed traditional ICG dye in terms of postoperative outcomes. Pathological studies of the resected tumor tissue indicated that the POH experimental group achieved more precise resection compared to traditional surgery and ICG guided resection. This highlights the advantages of semi-cyanine structures over traditional cyanine structures in the surgical guidance of gliomas.

In this study, we aimed to address the challenges of glioma surgical navigation by developing and evaluating a novel NIR-II viscosity responsive fluorescent probe, POH. Based on a semi-cyanine molecular rotor structure, POH was hypothesized to provide enhanced optical performance, tumor specificity, and intraoperative visualization compared to conventional cyanine dyes. We designed a series of experiments to examine the optical properties, viscosity-dependent fluorescence responsiveness, biosafety, and potential of POH for fluorescence-guided tumor resection in a preclinical glioma model.

## Methods

### Materials

Unless otherwise noted, all reagents were obtained from commercial resources and used without further purification. Tetrahydrofuran (THF), toluene (Tol), and dichloromethane (DCM) used for reactions were purified by evaporating after fully stirring with Na and using benzophenone as the indicator. All air and moisture-sensitive reactions were carried out in flame-dried glassware under a nitrogen atmosphere.

### Quantum yield test

The quantum yield (QY) of the fluorophores was measured using previously reported procedures with modifications (45). The NIR-II fluorescence emission intensities were measured under the same 808 nm excitation. The QY values of the samples were determined based on five concentrations with gradient optical densities (ODs) at 808 nm. Using the measured ODs at 808 nm and the integrated fluorescence intensity, the quantum yield of a test sample was calculated according to the following equation (45):


$$\varphi_{x}\left(\gamma \right)=\varphi_{std}\left(\gamma \right)\times \frac{F_{X}}{F_{std}}\times \frac{A_{std}\left(\gamma \right)}{A_{x}\left(\gamma \right)}\times (\frac{\eta_{x}}{\eta_{std}}{)}^{2}$$

$\varphi_{x}$: fluorescence quantum yield of the sample; $\varphi_{std}$: fluorescence quantum yield of the reference standard; *F*: spectrally integrated fluorescence intensity; *A*: measured OD at 808 nm; *η*: the refractive index of solvent.

### Photostability

5μM POH and ICG were dissolved in phosphate-buffered saline (PBS). The fluorescence signal was monitored using a two-dimensional InGaAs camera under continuous exposure to an 808 nm laser at a power density of 65 mW/cm^2^. The average fluorescence intensity of the region of interest (ROI) was plotted as a function of time.

### Cell viability assessment using the MTT assay

The 4T1, U87, C6, and L-02 cells (5000 per well) were seeded into a 96-well plate (NEST) and incubated for 12 h at 37 °C in a humidified incubator with 5% CO_2_. Then, DMEM solutions with 0.1, 1.0, 2.0, 5.0, and 10.0 POH were added. After 24 h of incubation, the cells were rinsed three times with PBS and 100 μL MTT (0.5 mg·mL^-1^) solution was added. After removing the MTT solution after 4 h of incubation, 50 μL DMSO was added to each well. Placing the shaking table at a low speed for 2 h allowed the crystal to dissolve fully. The absorbance value of each well was measured at OD 570 nm using an Elisa reader (BioTek Synergy LX).

### Breast tumor and glioma models

All animal experiments were conducted under institutional guidelines and were approved by the Experimental Animal Ethical Committee of the First Hospital of Jilin University (Protocol number 20210912). BALB/c mice were purchased from Liaoning Changsheng Biotechnology Co. Ltd. Bedding, nesting materials, food, and water were provided ad libitum. The ambient temperature was controlled between 20°C to 24°C. The 4T1 cells were cultured *in vitro* to the logarithmic growth phase and inoculated into the subcutaneous fat pad of the chest of female BALB/c mice (4–6 weeks old, body weight 18–20 g) at several 10^6^ cells per mouse (46). The successful model was established by palpable tumor nodules at the site of the subcutaneous tumor around 5 days after modeling. For the glioma model, C6 cells were cultured *in vitro* to logarithmic growth phase and then used to establish the model by using BALB/c Nude female mice (4–6 weeks old, 18–20 g body weight) at several 3×10^6^ cells per mouse (47). The mice were injected with 200μL cancer cells into the axillary fat pad of the upper limb using the free needle method. After 7 days of natural feeding, the subcutaneous tumor model was formed.

### NIR-II fluorescence imaging of 4T1 (breast) and C6 (glial) based murine tumor models

Under 808 nm excitation laser, 850 nm short-pass (SP) and 900/1000 nm long-pass (LP) filters were used to collect NIR-II bioimaging. POH in PBS (100 μM, 25 μL) was injected into the tumor (n = 3) inoculated by 4T1 or C6 cells. The NIR-II images were obtained at 0 min, 30 min, 1 h, 3 h, 6 h, 12 h, 24 h, 48 h, 72 h, and 96 h post-injection.

### H&E staining

After harvesting, all the tissues were fixed in 4% paraformaldehyde. These tissues were further dehydrated, embedded in paraffin, and sectioned into 3 μm thick slides. H&E staining was then performed according to the protocol of the H&E kit (Beyotime Institute of Biotechnology, Cat. No. C0105). H&E staining images of every tissue were acquired using the Nikon Eclipse 80i microscope.

### NIR-II imaging

All mice were shaved using Nair depilatory cream, anesthetized with chloral hydrate or isoflurane before the experiment, and placed on the imaging table. At least three mice were used as parallel controls in each imaging experiment. All NIR-I/NIR-II images were collected on a two-dimensional InGaAs array (Princeton Instruments, NIRvana-640) with a laser wavelength of 808 nm and a power density of 65 mW/cm^2^.

### Metabolism assessment

Under 808 nm excitation, 900 and 1000 nm long-pass filters were used to collect NIR-II imaging under the InGaAs camera. POH in PBS (100 μM, 200 μL) were intravenously injected into six-week-old BALB/c mice (n = 3). Images were obtained at 0 min, 30 min, 1 h, 3 h, 6 h, 12 h, and 24 h post-injection.

### NIR surgical navigation

A medical student was brought to perform this fluorescence-guided procedure. We performed this surgery and postoperative suturing and sterilization guided by signals from the NIR-II region. We considered a complete resection as the intensity of the NIR signal at an artificially defined site of interest was below a set value.

During this procedure, a 2×2×2 cm^3^ near the axillary fat pad of the mouse model establishing the tumor was specified as an artificial region of interest, and we observed the NIR-II signals of this region in the software accompanying the NIR camera after performing intratumoral injection of the probe. Combined with the results of the signal intensity experiments in the previous section, we defined the area with more than 50% of the signal as the “tumor area” and performed surgical resection. For the surgical navigation experiment, tumor-bearing mice were randomly divided into two groups (n=6 per group): the POH experimental group and the ICG control group. The optimal concentration of POH was first determined based on its fluorescence properties and biosafety profile. For comparison, ICG was adjusted to produce a similar fluorescence intensity under identical imaging parameters, and both probes were injected intratumorally with the same volume to ensure experimental consistency. This approach allowed a direct comparison of their fluorescence behavior within the high-viscosity tumor microenvironment. Near-infrared camera imaging conditions in surgery: Under 808 nm excitation, 900 and 1000 nm long-pass filters were used to collect NIR-II imaging under the InGaAs camera. At the end of the experiment, all mice were sacrificed by cervical dislocation after isoflurane anesthesia.

## Results and discussion

### Selection and validation of viscosity-responsive probes

ICG (Indocyanine Green) was the first cyanine-based drug approved by the FDA for angiography (48–51). The fluorescence intensity increases when ICG binds to proteins has made it widely popular in various clinical applications. Beyond angiography, ICG is also extensively used in clinical surgical guidance (27, 50, 52, 53). Although ICG has demonstrated significant efficacy in many clinical studies (26, 54, 55), gliomas pose unique challenges compared to other tumors. Gliomas are typically located in deep brain structures and are responsible for critical functions such as motor skills, sensation, language, and cognition. This makes the resection of gliomas distinct from other tumors, requiring extreme caution with surgical margins (1–3). Additionally, gliomas exhibit high invasiveness with often unclear tumor boundaries, making complete resection without damaging healthy tissue is challenging and increasing the risk of tumor recurrence. Thus, exploring clearer visualization techniques for glioma surgical guidance is crucial.

**Figure 1:**
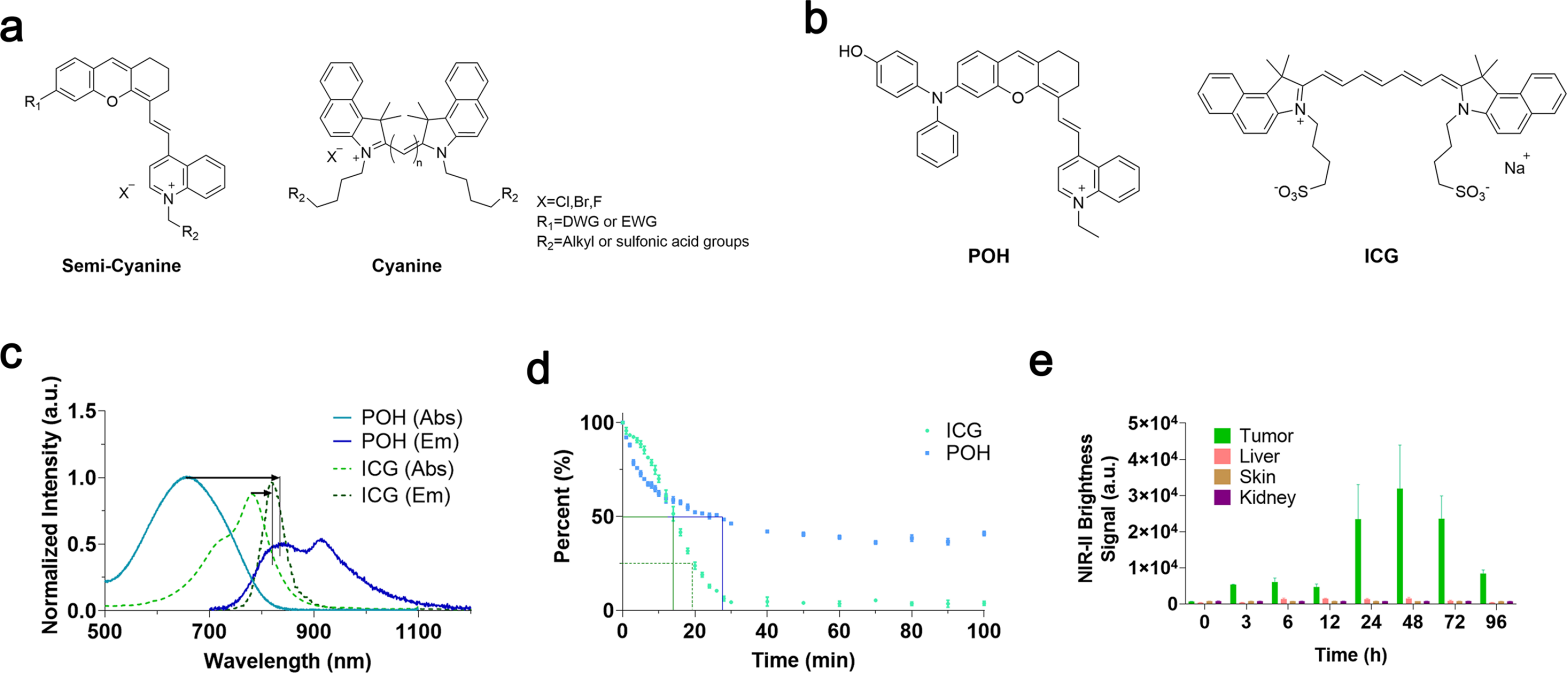
Optical structure advantages and tumor responsiveness of semi-cyanine dye. **(a)** Schematic illustration of the chemical structures of semi-cyanine and cyanine-based NIR dyes. **(b)** Chemical structures of representative semi-cyanine dye POH and cyanine dye ICG. **(c)** Absorption/emission spectra of POH and ICG (testing conditions: 10 μM probes in DMSO, excitation wavelength 700 nm). **(d)** Comparison of photostability between POH and ICG. **(e)** Time-dependent statistical values of *in vivo* NIR signals post-intratumoral injection of POH probe in 4T1 tumor model (injection dose: 25 μg/cm³, imaging conditions: 808 nm excitation, LP = 900 + 1000 nm, exposure time 200 ms).

Addressing glioma-specific challenges, we selected a class of near-infrared semi-cyanine dyes with classic molecular rotor structures, as shown in **Figure 1a**, due to their viscosity-responsive properties. Recently reported, the viscosity-responsive probe POH was chosen as the experimental group to be compared with classic ICG (**Figure 1b**). This structure features a classic molecular rotor and a positive ion structure for targeting organelles. Optically, the Stokes shift of POH is 178 nm, significantly larger than ICG’s 36 nm, which reduces dye photon reabsorption, minimizes background signal interference, and allows for potential multi-channel surgical navigation (**Figure 1c**). The quantum yield of POH in PBS was determined to be 0.08–0.008% in the NIR region using IR-26 as a reference. Moreover, POH shows much higher photostability than ICG (**Figure 1d**). For clinical surgeries lasting 1–2 hours, a more stable and irradiation-resistant dye is undoubtedly more practical.

To confirm the viscosity-responsiveness of POH, we conducted two experiments (56). First, fluorescence intensity was measured in a glycerol–water system with viscosities ranging from 0.89 to 1490 cP, showing an approximately 80-fold increase in signal with increasing viscosity. Second, POH was tested in the presence of common inorganic ions and glutathione, where no significant fluorescence changes were observed; fluorescence enhancement occurred only in glycerol-rich (high-viscosity) media. These results demonstrate that POH is selectively activated by viscosity rather than nonspecific chemical factors, confirming its function as a viscosity-sensitive probe.

The 4T1 breast tumor model was first used in the preliminary stage to evaluate the fluorescence characteristics, biosafety, and metabolic behavior of POH under a convenient and reproducible subcutaneous tumor setting, prior to its application in the glioma model for surgical navigation studies. We then used POH for intratumoral injection in 4T1 tumor-bearing mice. No substantial signal increase was detected in vital metabolic organs such as the liver and kidneys (**Figure 1e**). Statistical analysis revealed minor near-infrared fluorescence in the liver and abdomen during metabolism, suggesting that after intratumoral injection, the dye undergoes classical hepatobiliary metabolism and is excreted via feces. Dissection of mice at key time points confirmed near-infrared fluorescence predominantly in the liver (**Supplementary Information**, **Supplementary Figure S1a**). Histological examination of major organ cross-sections stained with Hematoxylin and Eosin (H&E) showed no significant organ damage (**Supplementary Information**, **Supplementary Figure S1b**). Collection of excreta from injected mice revealed notable near-infrared fluorescence in feces (**Supplementary Information**, **Supplementary Figure S2**), supporting our hypothesis regarding the metabolic pathway, consistent with previous literature reports.

### Evaluation of responsiveness and biosafety in glioma model

**Figure 2:**
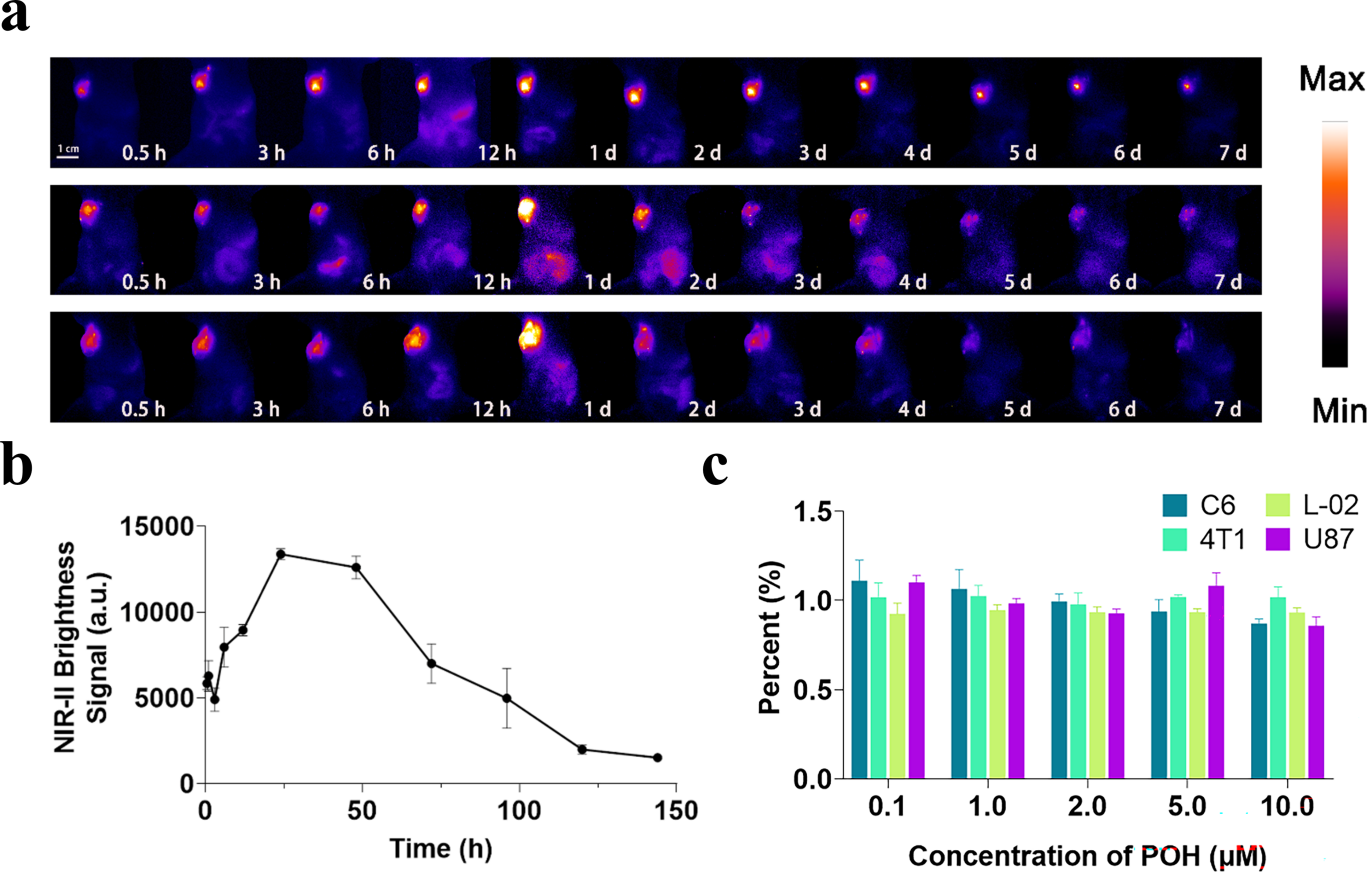
Responsiveness verification of POH in C6 mouse tumor model. **(a)** NIR-II *in vivo* imaging of multiple tumor model mice at key time points post-intratumoral injection (injection dose: 25 μg/cm³, imaging conditions: 808 nm excitation, LP = 900 + 1000 nm, exposure time 200 ms). **(b)** Statistical graph of NIR signal intensity at the tumor site over time. **(c)** Cell viability of various cells (C6, L-02, 4T1, U87) at different concentrations of POH.

To investigate the performance of this material in gliomas, we established subcutaneous C6 tumor models and conducted intratumoral injections at the same dosage (**Figure 2a**). Near-infrared camera tracking of key time points post-injection indicated an optimal response signal around 24 hours (**Figure 2b**). This differs from the optimal time point observed in the 4T1 model, likely due to variations between different tumor models. Statistical analysis of the NIR-II signals at the tumor sites (outlined manually as shown in the figure) revealed that in most C6 glioma model mice (n = 6), the fluorescence signal decreased to less than 5% of the maximum within seven days post-injection.

This strongly suggests that the probe can be adequately metabolized post-intratumoral injection and that this metabolic behavior is highly reproducible. Dissection and organ extraction from the metabolism experiment mice showed distribution patterns similar to the 4T1 model, with primary concentration in the liver (**Supplementary Information**, **Supplementary Figure S3a**). H&E staining of these organs showed no significant tissue damage, and the probe exhibited reasonable safety profiles in different cell types (**Figure 2c**). Overall, the probe demonstrated good biocompatibility (**Supplementary Information**, **Supplementary Figure S3b**).

### NIR fluorescence-guided surgical navigation and outcome evaluation

**Figure 3:**
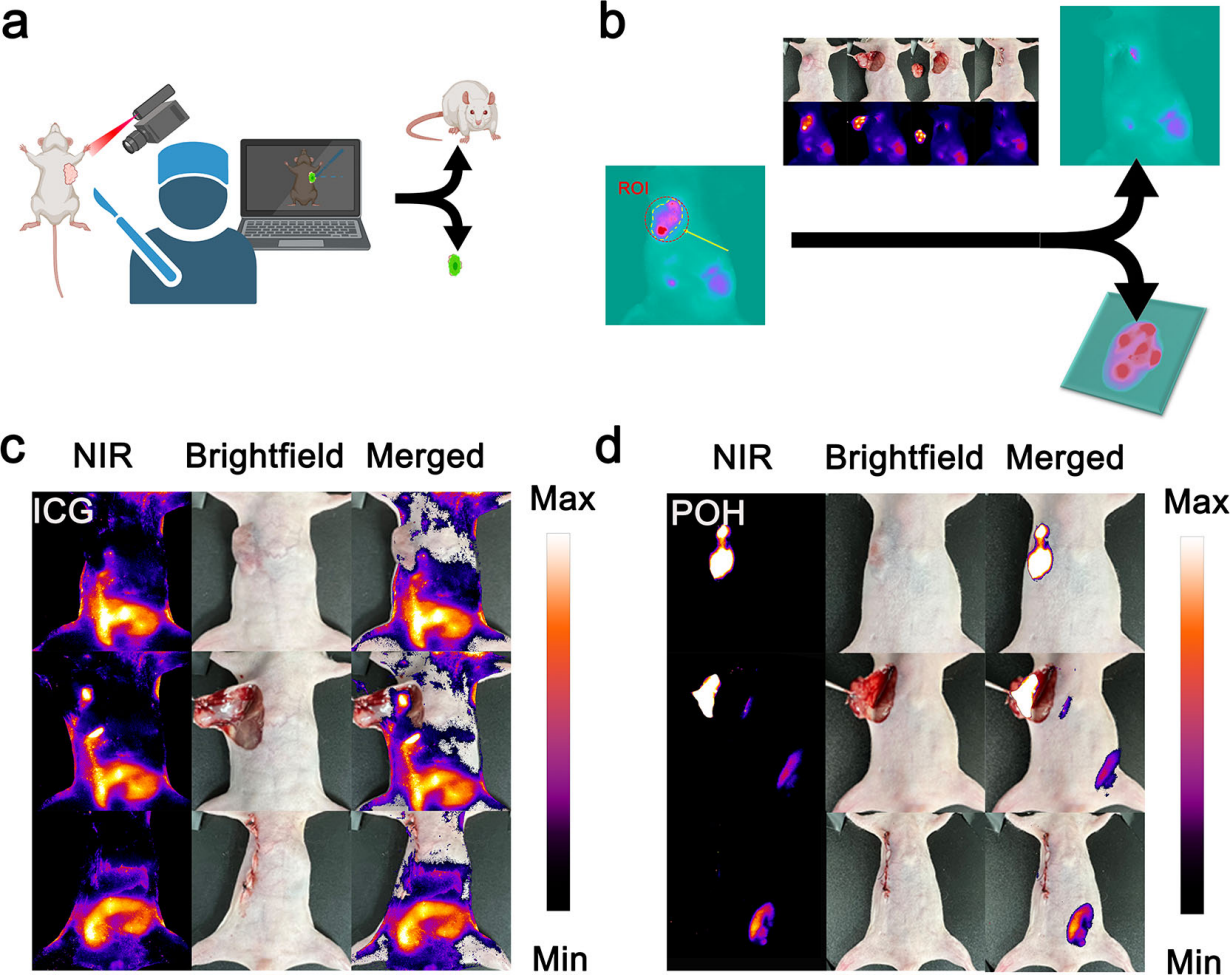
NIR fluorescence-guided subcutaneous tumor resection surgery. **(a)** Schematic diagram of the NIR fluorescence surgery navigation system. **(b)** Determination of the resection area (region of interest circled in yellow) and post-resection results. **(c)** Key resection steps in the ICG group and **(d)** POH group, showing bright-field, NIR fluorescence, and overlay images (imaging conditions: 808 nm excitation, LP = 900 + 1000 nm, exposure time 200 ms).

Subsequently, we enlisted clinically experienced medical students to perform surgical resection of tumors in C6 model mice, guided by NIR fluorescence signals visible in the camera’s field of view (**Figure 3a**). Tissue exceeding the fluorescence signal threshold within the delineated area was removed (criteria for signal determination are detailed in the supporting information). As shown in **Figure 3b**, we present the surgical process’s bright-field and NIR fluorescence images, highlighting four key stages: preoperative delineation, excision, complete removal, and suturing. The probe POH used in the experimental group, and the probe ICG used in the control group were normalized based on fluorescence signal intensity before injection. For both POH and ICG, intratumoral injection was employed to ensure identical local probe concentrations and to directly compare their fluorescence responsiveness within the same tumor microenvironment. Although ICG is typically administered intravenously in clinical practice, intratumoral delivery is a commonly used experimental method in preclinical imaging studies to evaluate localized fluorescence signal performance and eliminate systemic pharmacokinetic variables (57–59). The surgery was performed 12 hours post-injection for POH and 2 hours post-injection for ICG, corresponding to the optimal time windows determined in preliminary experiments. As seen in **Figure 3c**, the ICG group exhibited low signal intensity post-injection, particularly after skin incision, which was not significantly different from the preoperative signal. This indicates that ICG does not reliably provide preoperative information for the affected area. In contrast, POH demonstrated clearer fluorescence signal contrast and improved visualization of tumor margins both before and after tumor tissue removal (**Figure 3d**).

**Figure 4:**
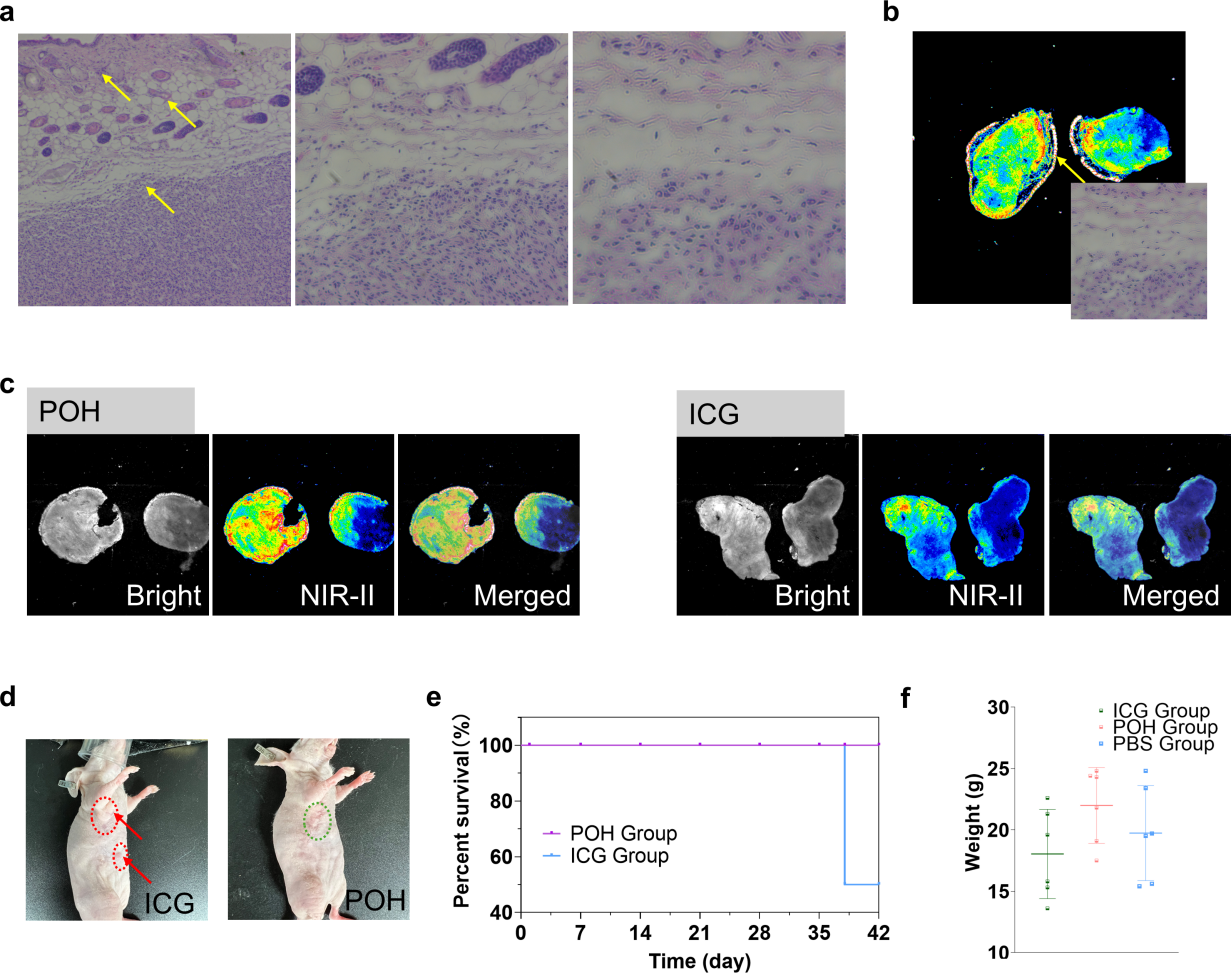
Pathological analysis of tumor tissue excised under near-infrared surgical navigation. **(a)** The H&E-stained section from the POH experimental group shows the tumor tissue margin. **(b)** NIR image of the section from the tumor tissue excised under NIR-II guidance. **(c)** Bright-field and NIR-II images of the tumor tissue excised from the POH and ICG experimental groups. **(d)** Bright-field images of mice from the ICG and POH experimental groups 28 days post-tumor excision surgery (red outlines indicate tumor recurrence areas). **(e)** Postoperative survival rates of mice in both experimental groups within 42 days after tumor surgery (n=6). **(f)** Weight changes in mice from the ICG and POH experimental groups 28 days post-tumor excision surgery.

We conducted further analysis of the excised tumor tissue structure to evaluate the experimental effects of the clinical surgery. The excised tumor tissue was embedded, sectioned, and stained with H&E, revealing a clear presence of large plasma cell nuclei and fibrotic structures at the tumor boundary and infiltration area (**Figure 4a**). The location of the sections used was sampled under the near-infrared camera, as shown in **Figure 4b**. We analyzed the excised tumor tissues from the two experimental groups mentioned earlier, observing their section signal distribution under the near-infrared camera (**Figure 4c**). Evidently, the section signals in the POH experimental group were significantly stronger than those in the ICG group. The signal at the tissue boundary was notably intense, which is highly beneficial for guiding clinical surgical procedures. This further demonstrates the precision advantage of POH as a surgical navigation probe.

Additionally, we monitored the experimental mice’s postoperative progress for six weeks, recording their weight and physical condition. Among the six mice in the experiment, the ICG group exhibited tumor recurrence in the third to fourth weeks, leading to mortality by the sixth week (**Figures 4d, e**). Throughout the postoperative recovery period, no significant differences in weight were observed among the mice (**Figure 4f**).

While our findings support the use of POH for enhanced intraoperative visualization, we acknowledge that the current evaluation of surgical precision remains qualitative, based on fluorescence imaging and histopathological analysis. Quantitative volumetric methods—such as calculating the percentage of tumor volume resected, assessing residual tumor mass, and measuring margin clearance distance—were not employed in this study due to the limitations of the subcutaneous tumor model. In future work, we plan to utilize orthotopic glioma models in combination with MRI or CT imaging to enable three-dimensional reconstruction and objective evaluation of resection completeness. This will allow for a more rigorous assessment of POH’s surgical navigation performance.

This study did not assess POH signal behavior in non-tumor inflammatory conditions such as LPS-induced brain edema. Although POH activation is governed by microviscosity rather than nonspecific inflammatory cues, future work should include inflammatory and ischemic brain models to comprehensively evaluate probe specificity and exclude potential false-positive signals.

### Limitations of the study

Despite the promising results presented in this study, several limitations should be considered. First, while the viscosity-responsive probe POH demonstrated superior tumor targeting and imaging contrast in the C6 glioma model, its efficacy and safety in human patients have yet to be fully evaluated. It should be noted that an additional 4T1 breast tumor model was also used in the preliminary phase to assess the probe’s fluorescence behavior and biosafety, but the primary fluorescence-guided surgery experiments were conducted in the glioma model. Clinical translation of NIR-II probes like POH requires rigorous testing, including long-term toxicity studies, pharmacokinetics, and pharmacodynamics evaluations. Second, our study was conducted using a limited number of preclinical models, primarily focusing on C6 glioma-bearing mice. While these models are commonly used in glioma research, they may not fully replicate the heterogeneity and complexity of human gliomas, particularly with regard to their response to surgical intervention and fluorescence guidance. Furthermore, a notable limitation of our study is the use of subcutaneous glioma models rather than orthotopic (brain) glioma models. Subcutaneous implantation of glioma cells does not accurately replicate the clinical behavior of gliomas, as it lacks the unique microenvironment of the brain, including the blood-brain barrier, tissue stiffness, and the complex tumor-stroma interactions present in native glioma tissue. Although subcutaneous models are useful for initial proof-of-concept studies, future research should incorporate orthotopic glioma models to better assess the probe’s targeting and performance in a setting that more closely resembles human gliomas. This would enable a more accurate evaluation of POH’s clinical applicability, particularly in the context of brain tumor surgeries.

## Conclusion

This study evaluated a viscosity-responsive NIR-II fluorescent probe, POH, for fluorescence-guided glioma surgery in a preclinical model. POH demonstrated favorable optical properties and improved visualization of tumor margins compared to ICG. These findings support its potential as a candidate for guiding glioma resection.

A key limitation is the use of subcutaneous rather than orthotopic models, which do not replicate the brain tumor microenvironment. Future studies will employ orthotopic glioma models to better assess clinical relevance. In addition, surgical completeness was assessed qualitatively. Future research will incorporate quantitative imaging methods to validate resection outcomes more objectively.

## Data Availability

The original contributions presented in the study are included in the article/**Supplementary Material**. Further inquiries can be directed to the corresponding author.
