# Supplementary material for: A novel near-infrared II viscosity-responsive probe for surgical fluorescence guidance: laboratory investigation in a murine subcutaneous glioma model
Source: Front Oncol. 2026 Feb 11;16:1586263. doi: 10.3389/fonc.2026.1586263 (PMC12932158; doi:10.3389/fonc.2026.1586263)
Supplement: Supplementary file 1 [file DataSheet1.docx]

Supporting Information

A novel near-infrared II viscosity-responsive probe for surgical fluorescence guidance: laboratory investigation in a murine subcutaneous glioma model

Lihao Lin, Tianyang Han, Huizhong Jiang, Yuewei Zhang & Yi Guan*

1. Synthesis and characterizations of POH
2. Supplementary figures

**1. Synthesis and characterizations of POH**

The related synthesis data has already been disclosed in our another work. Here, we only present the structural purity data of the probes used.

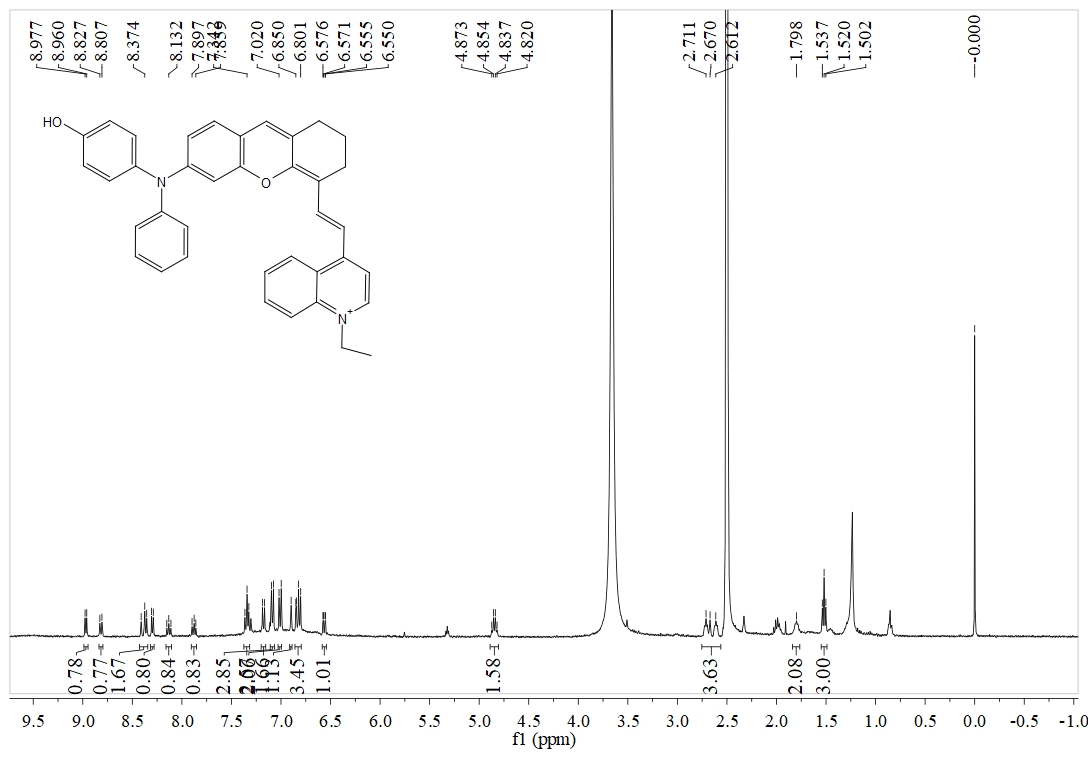


**2. Figures**

**
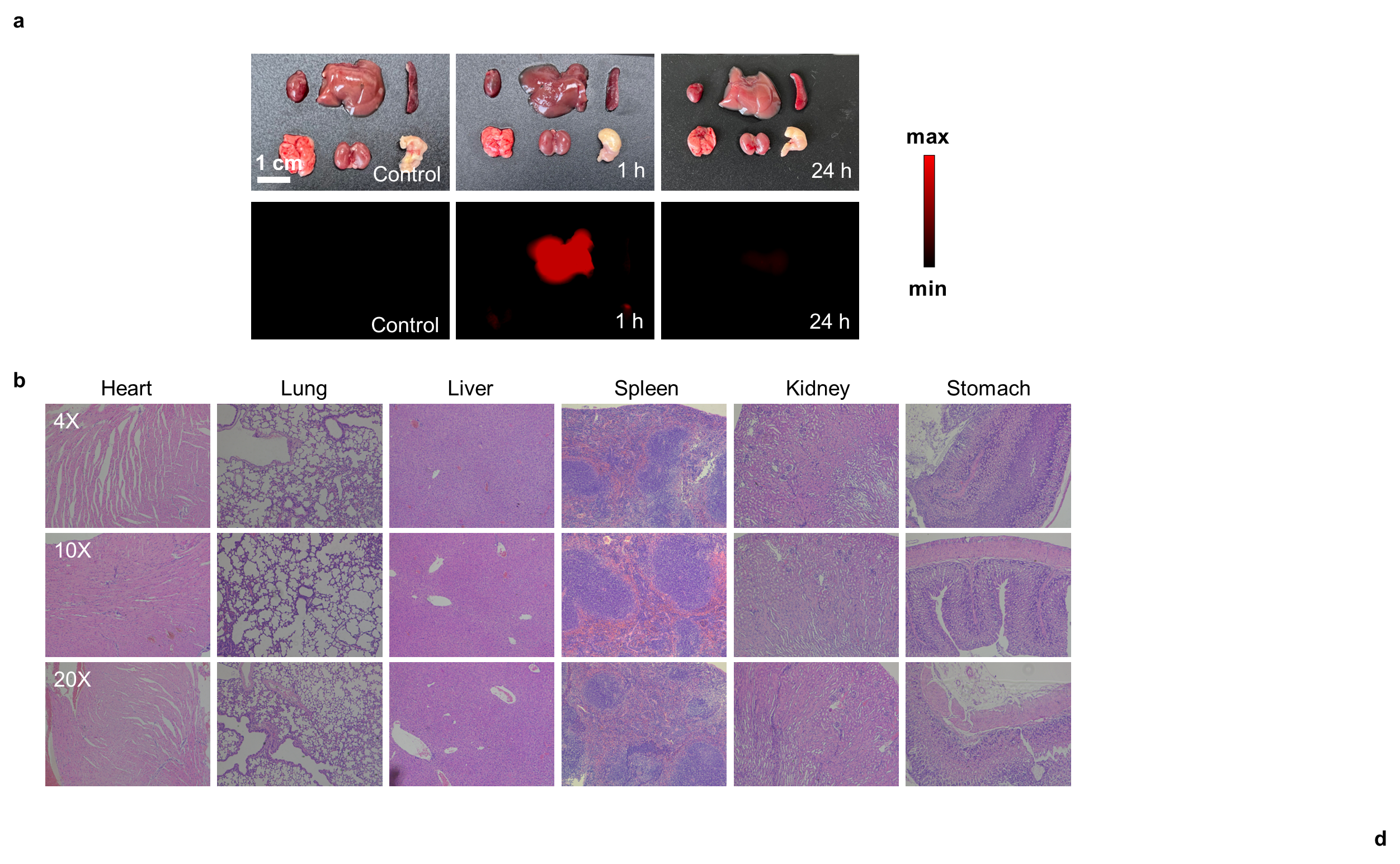
Fig. S1. Biosafety assessment of POH. a**) NIR-II fluorescence images (lower) and photographs (upper) of organ anatomy (heart, liver, spleen, kidney, lung, and stomach) from the control and POH intravenous administration groups at 1 and 24 h. **b**) Histological evaluation of main organ anatomy after intravenous administration of POH.

**
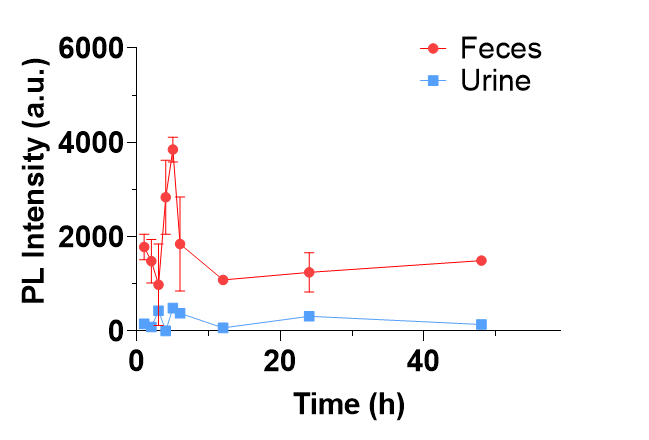
**

**Fig. S2. Fluorescence intensity of the collected feces and urine after intravenous injection of POH at relevant time points.**

**
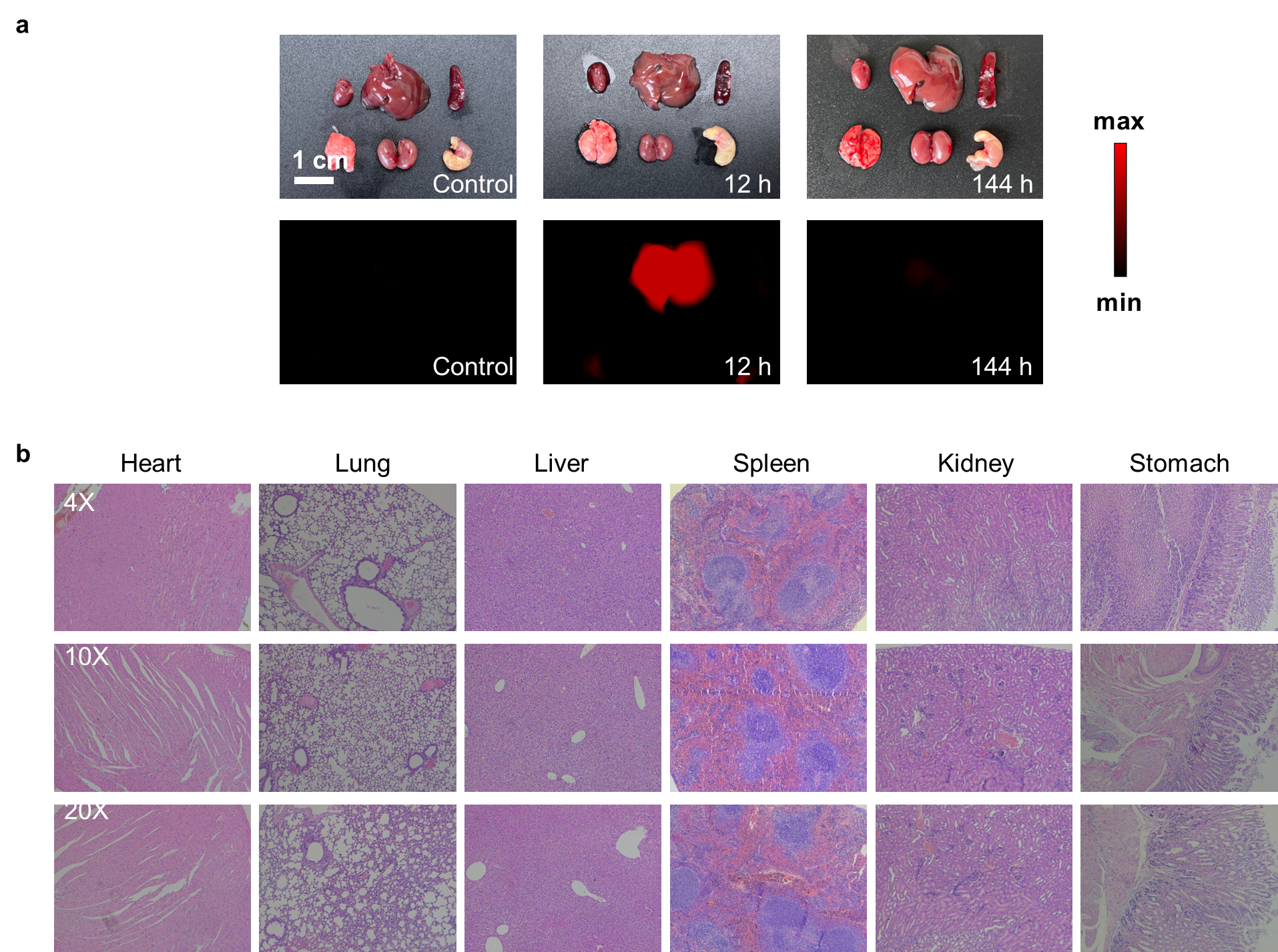
**

**Fig. S3. Metabolism assessment of POH in C6 Model. a**) NIR-II fluorescence images (lower) and photographs (upper) of organ anatomy (heart, liver, spleen, kidney, lung, and stomach) from the control and POH intravenous administration groups at 12 and 144 h. **b**) Histological evaluation of main organ anatomy after tumor imjection of POH.
